# Running as a Form of Therapy Socio-Psychological Functions of Mass Running Events for Men and Women

**DOI:** 10.3390/ijerph15102262

**Published:** 2018-10-16

**Authors:** Ewa Malchrowicz-Mośko, Joanna Poczta

**Affiliations:** Department of Cultural Foundations of Tourism, University School of Physical Education in Poznan, 61-871 Poznan, Poland; malchrowicz@awf.poznan.pl

**Keywords:** motives for running, women in sport, men in sport, half marathon, runners, non-elite sport

## Abstract

The main goal of this study was to recognize the motives of participation in mass running events (Poznan Half Marathon case study). The second aim was to examine the motivations to participate in a half-marathon among two groups of respondents: men and women, and to evaluate the differences between them. The empirical research among runners (*n* = 560) conducted during the one of the most important running events in Poland recognized the motives for participation in the sports event in accordance with the four basic types of orientation: social orientation, experience orientation, factual orientation, and result orientation (Freyer & Gross, 2002). The significant difference between surveyed men and women appeared in the motives of desire to get away from everyday life and its prevailing fashion, which turned out to be more important for women than for men. The desire to win was not important for the respondents. All respondents reported that what was very important for them was the need to experience strong emotions related to participation, the desire to feel unity and integration with other people, and the desire to test themselves. In general, the results show that people participate in running events not only for physical activity, but also for mental well-being and socio-psychological effects. They experience strong emotions, adrenaline, pleasure, relaxation, and an escape from the duties and hardships of everyday life and have an opportunity to build social relationships thanks to mass sports events organized in urban agglomerations. The article constitutes a conceptualization of the running event in the dimension of social and psychological interactions, which reveal and demonstrate its creative layers and contemporary meaning which has already gone deeply beyond functions of running for health and improving the physical condition.

## 1. Introduction

In today’s world, people look for the possibilities for developing their passions and skills also in the area of entertainment and leisure time. They are searching for (as predicted by Stebbins, 1982) “the world of leisure for ways to express their abilities, fulfill their potential, and identify themselves as unique human beings” [1]. Serious leisure theory is current nowadays. This phenomenon is describes as a main motivation for people with these goals. Its three types—amateurism, hobbyist pursuits, and career volunteering—are interrelated and opposed throughout with unserious or casual leisure, and work. The subsidiary position of serious leisure between these two extremes demotes its current participants to the status of minor importance men and women of leisure [1]. Since the 1960s, the “running sport” has been growing internationally as part of a specific trend which is defined as “desportification and deinstitutionalization of the sport sector”. This trend implies that adults no longer engage in sports because of the competitive aspect, but mainly because sports are first of all healthy, relaxing, adventurous or pleasant [2,3,4,5,6]. People are searching for situations posing an ability to verify their own achievements, constantly checking their progress, reaching a high level of stimulation and emotion. The sense of a high quality of life and the sense of pleasure resulting from an active lifestyle are determined not only by the way of motivational processes, but also by the level of satisfaction of psychological needs which are a precondition for reaching satisfaction from one’s own physical activity [7]. This occurrence has made participation in organized sports events an extraordinarily attractive form of physical recreation, practiced by an increasingly growing number of sports enthusiasts. People often want to become amateur athletes and take on the challenges of a sporty lifestyle. They look for strong emotions provided by sports rivalry, and look for opportunities to establish ties and social relationships (the desire to feel the sense of community during the events). They eliminate anxiety, depression, mood changes, they feel free and healthy, and they improve their quality of life. This phenomenon describes very well R. Crawford’s theory of healthism—a lifestyle planned with a view of health and fitness, based on personal pursuit and building motivation to reach the expected health goals. He successfully explains that healthism also appeals to the way in which we treat social and moral values related to health. This process is closely related to the habits of everyday life and how people perceive themselves and the condition of their health against their fellows and the social environment. According to Crawford [8,9,10], healthism represents a certain high value for many citizens in developed economies and involves the application of numerous social practices and solutions that aim at strengthening or even improve the individual’s health and to encourage others to do the same. In his concept concerning health he states that since the 1970s public health awareness has been a consequence of political ideology [8,9,10,11,12]. Public healthcare systems of the 21st century in many countries are more closely related to market mechanisms. Many countries have withdrawn from public health insurance, which means that citizens should be aware of their responsibility for controlling their health and well-being [13]. In addition, the public sector provides access to a wide range of health information and is able to manage the “consumer” and their rational choices [14,15,16,17,18,19]. Crawford argues that “solving health problems and taking care of health depends on individual determination, selection skills, and resistance against what results from culture, advertising, institutional and environmental constraints, work of medical representatives, or simply laziness and own habits” [8]. The ideology of healthism has been developing in Poland, too. One such practice is the promotion of widespread sport. This trend is especially visible in mass running events, in which Poles participate willingly. The range of this social phenomenon is an extreme and unusual occurrence on many levels. That is why a number of interdisciplinary components should be taken into account. However, as Crawford noted, health should be achieved through self-effort [7].

In health research, there has been an ongoing discussion about the potential of running events to increase overall sport participation and physical activity among adults [20]. Organizers of running events as well as the sport and recreation industry in general, claim there is an exercise-enhancing effect of participating in such events, but empirical evidence supporting such claims is not very consistent [21]. Some studies carefully confirm a potential positive effect on physical activity [20,21,22,23], but long-term effects have rarely been examined [22]. Furthermore, potential positive effects seem to be dependent on socio-demographic factors, motivation, and prior physical activity levels [21]. The research into the influence of the organization (broadly) of sporting events and of running events on various aspects of the lives of the participants was carried out by many researchers. Hardly anyone examined and distinguished the impact of running-event participation in terms of gender. For example, Hover and co-authors [24] analyzed the creation of the social impact of sports events (environmental, economic, and social indicators of undertaken physical activity), proving that it is very significant and generally positive. Biddle [25] examines the impact of sport on the psychosocial condition, providing its positive impact on people. But gender in this context was interesting for Maier [26] who divided the impact of sport on people according to gender, just as in the Polish scientific environment. Waśkowski [27] studied the motives, preferences, and expectations of women towards running, and Stępień [28] analyzed the experience of women’s participation in the marathon and made an attempt to determine the importance of the participation in marathon races and the role of running as a leisure-time activity in their lives. However, the main theoretical approaches to studying the motivations for undertaking sport are based on specific psychological theories as well as methods based on the concept of “achievement motivation” and the concept of doing sport as a “positive addiction” [29]. Masters and co-authors [30] began to develop a questionnaire The Motivations of Marathoners Scales (MoMS), to measure motivation for marathon running, including distinguishing motivations connected with gender. According to the authors of the questionnaire, the study of the qualitative aspects of motivation of marathon runners for running marathons was the first step to explore the diversity of motives for running [30]. The research of Masters and coauthors showed satisfactory psychometric indicators, defining the reliability and accuracy of the questionnaire, as well as the adequacy of the model. So far the MoMS scale has been used in many studies, for example the ones exploring motivation of marathon runners for running marathons due to: the level of experience [31], gender [32], cognitive strategy [33] and age [34]. The study of Ogles and co-authors [32] from 1995 (*Obligatory running and gender: an analysis of participative motives and training habits*) compared the participative motives of male and female runners registered in races of varying lengths (marathon, half-marathon, and 5 K/10 K) using the Motivations of Marathoners Scales. The main results show that women endorsed weight concern, affiliation, self-esteem, psychological coping, and life meaning as more important motives for running than men. In addition, a select group of behaviorally identified obligatory and recreational runners were compared in terms of self-reported motivations for running. Men were disproportionately identified as obligatory runners. Male obligatory runners are characterized by an emphasis on achieving recognizable success. That is, obligatory runners more heavily endorsed achievement motives (competition and personal goal achievement) and recognition as reasons for training. In contrast, male recreational runners more heavily endorsed physical well-being motives (general health orientation and weight concern) as reasons for training. The obligatory runner runs excessive miles per week, running despite injury or other personal costs, guilt, nervousness, suffers from depression and other withdrawn-like symptoms when not running, never takes days off etc. [32]. Yates and co-authors [35,36,37] argue that running is an analogue of anorexia for men and that men are more likely to become obligatory runners. Yates [37] also concludes that women tend to be evaluated on their physical attractiveness while men tend to be evaluated of their physical effectiveness or strength. Summers and co-authors [38] claim that women report greater benefits from running than men in terms of opportunities to meet people, relief from depression, and feeling less shy. Authors Callen [39] and Barrel [40] proved that men tend to train more miles but Summers [38] proved that women report higher levels of addiction to running. Researchers Harris [41] and Masters with co-authors [30] indicated that women are also more likely to report initiating and continuing to run as a way of controlling their weight and staying fit. The fashion for running in Poland is now at its highest level. This prompts the question as to why a postmodern person so willingly and so often participates in such sports events, especially since some research in this field has already been around for several years.

The inspiration to carry out this research was not only the growing popularity of running in Poland but also the fact that there are no current research results on the differences between the motives of participation in running events between men and women. The idea to carry out the research came from the Freyer and Gross theory [42]. The authors generally distinguished four main types of orientation among the motives for participation in sports events: (a) social orientation, focused on the relationships of sport-lovers to each other; (b) experience orientation, most often referring to the positive experiences, in the form of, for example, relaxation, which is a kind of compensation for the hardships of everyday life; (c) factual orientation/sports discipline orientation, concerning the sports events themselves and to their specificity; in this case, to the specificity of the sport which is running; (d) result orientation, triggered by the need to identify with success, and in the case of failure, by the need to show sympathy and solidarity. The division of Freyer and Gross was the basis for the development of the authors’ questionnaire survey of motives for participation in running events and was used over the 6th Poznan Half Marathon, to recognize the motivations to participate in a half-marathon among two groups of respondents—men and women—and also to evaluate the differences between them and determine the impact of their participation in the running event on their mental health. The impulse to verify these differences was the respondents’ different sex, lifestyle, needs, and motivations for the involvement in sport. Moreover, the research area was also an inspiration, because more than 500 running events are organized annually in the city of Poznan and in Great Poland region, which places the city between the leaders of mass running events all around Poland.

## 2. Aims of Study

The main goal of the study was to recognize the motives of participation in mass running event. The second aim was to examine the motivations to participate in a half-marathon among two groups of respondents: men and women, to evaluate the differences between them. Diagnosis of the motives for participation in sports event has been examined in accordance with the four basic types of orientation: social orientation, experience orientation, factual orientation and result orientation in order to check what benefits for mental well-being, good mood and good self-improvement are brought by the participation in the running event.

## 3. Materials and Methods

### 3.1. Design and Participants

In Poland, the number of organized events and their participants has grown noticeably since 2000. In 2013, there was a significant increase in the organization of mass running events. For example, in 2003 518 running events were organized, when in 2013 there were 2700 such events throughout the country [43]. As reported by Waśkowski [44], in the 12 largest running events in Poland the number of participants increased sixfold during a three–year period (2011–2013). Poznan is an example of a city with a population of over 500,000, and with a very wide range of sports events on offer. More than 500 events at various levels are organized annually in the city and the Wielkopolska region, which puts Poznan among the leaders of running events nationally. The Poznan Half Marathon is one of the biggest running events in the city and the whole country. The popularization of this sport and active participation in running events is multifaceted. Its most significant components concern health care, and physical and mental health. The sense of gaining a high quality of life and the sense of joy resulting from active lifestyle are conditioned not only by the course of motivational processes, but also by the level of satisfaction of psychological needs which are a prerequisite for achieving satisfaction from one’s own physical activity. People want to become amateur athletes and take on the challenges of a sporty lifestyle. The research was conducted to assess reasons for attempting a half marathon, the perceived outcomes from running a marathon, and participants’ experiences while running, including the production of feelings of deep personal awareness and satisfaction. The question was—what caused this “positive addiction”? That is why the authors decided to do the research by the diagnostic survey method using standardized interview technique during the 6th Poznan Half Marathon.

We tried to make the sample selection in a way that ensured the best possible representativeness of the results obtained. The scheme of simple random sampling without replacement was used. In determining the number, evidence from the organizers on the expected number of participants of the event was used. In calculations the formula for sample size for finite population was used. The assumption was made that the maximum error of estimate (e) at 95% confidence level should not exceed 4%.

A sample of 560 runners: 346 men respondents and 214 women participated in the event voluntarily and completed a questionnaire. The participants (*n* = 560) were mainly between 26–35 years old (40.7%—228) and 19–25 years old (40.2%—225). A smaller group were 36–50 year-old people (*n* = 69, 12.3%) and 51–70 year-old people (*n* = 25, 4.5%). Among the surveyed participants, people with higher education constituted the vast majority of 47.8% (268), 28.4% (159) possessed secondary education, and 19.1% (107) were people with incomplete higher education. A greater percentage of them—56.8% (318) were professionally active, and over 31.8% were students (178). The socio-demographic characteristics of respondents are presented below (Table 1).

Among the surveyed men (*n* = 346), the majority of runners were aged between 26 and 35 (40.8%—141), then between 19 and 25 (39.3%—136). Among the men, people with higher education constituted the vast majority of 46.8% (162), 29.8% (103) possessed secondary education, and 19.4% (67) were people with incomplete higher education. A greater percentage of them—58.4% (202) were professionally active. Most of the women (89) were aged 19–25 (41.6%). Among the surveyed women (*n* = 214) running in 6th Poznan Half Marathon, 106 completed higher education level (49.5%), 26.2% were women with secondary education, and 21% with incomplete higher education (Table 1). The employment statues defined by the female respondents was very good, because over 54.2% (106) of women participants were professionally active, over 34.1% were students (73). Socio-demographic characteristics in both groups of respondents (men and women) are similar.

### 3.2. Instruments and Procedure

A self-constructed questionnaire was used for the study, conducted (by the authors of this article) when the runners were finishing the race and personally filled out during the conversation with the runners. The results concerning a different type of motives (by the classification developed by Freyer & Gross) consisted of more than 100% because, in each group of motives, participants could tick more than one answer (maximum 3 answers). The questionnaire had 25 questions. The first part of the questionnaire focused on socio-demographic variables (Table 1). Questions about motives of participating in sporting events formed the second part of the survey. The third part of the questionnaire was designed for people who were residents of Poznan, and the last part was designed for sport tourists. For the purpose of the study, we have focused only on two parts of the questionnaire (first and second). The research instrument was validated before the examined event—during the 5th Poznan Half Marathon.

### 3.3. Analysis of the Data

Descriptive statistics (percentages, means and standard deviations) were calculated. For the differences between responses a Chi-square test for independence was used. Statistical significance was set at *p* < 0.05. We checked “Cramer’s Phi φ” (Effect Size for Chi-square Test) as well (in the case of statistically significant results). All statistical analyses were conducted using Statistica Software 10.0 (StatSoft Inc., Cracow, Poland, 2011).

## 4. Results

The analysis of all surveyed athletes shows that in the scope of social orientation motives (Table 2), the main factor for taking part in the half-marathon was the desire to feel unity and integration with other people—50.2% (281 runners). Belonging to the subculture of runners was on the second place—28.6% (160). The desire to gain recognition in the eyes of others (prestige) was important for every fifth competitor (21.8%). A similar percentage of respondents—21.2%—marked that they wanted to feel equality with other people during the race. For more than one in ten respondents, an important factor for taking part in the event was also the prevailing fashion—the fact that participation in sporting events in the field of mass sport is currently fashionable (12.5% of respondents).

The desire to experience strong emotions associated with participation was the most important motive in the Group B—motives within the scope of experience orientation—such an answer was declared by 65.2% (365) of the respondents. The desire to have good fun was important for 53.6% of surveyed athletes (300). The desire to feel the unusual mood during the whole event was declared by 48.4% (271) of the respondents. The desire to have enjoyable leisure was important for more than every fourth respondent—26.6% (149)—and the desire to get away from everyday life was important for over one in five athletes (21.3%). But almost 17.3% (97) of respondents felt the desire to express happiness, e.g., resulting from winning/success. Every twentieth competitor in the half-marathon was attracted by the city of Poznan (5.5%).

Table 3 demonstrates motives within the scope of factual and result orientation. Almost three-quarters of the competitors—70.9%—wanted to develop their passion for running. Over a quarter of competitors (27%) came to Poznan due to the attractiveness of the sports part of the half-marathon (including an attractive route). Every tenth athlete—11.3%—also took part in the event because of the attractiveness of the extensive program of accompanying events.

Nearly three-quarters (418) of the surveyed athletes—74.6%—expressed their willingness to test themselves. More than a half—59.6%—expressed their desire to achieve the avowed goal (336). Participation in sports competition was important for only 38.8% of respondents. 3.4% of athletes noticed the high, international rank of the half-marathon in Poznań, and only 2.1% of respondents declared that the motive for taking part in the half-marathon was the desire to win.

The desire to maintain physical condition and health was important for 460 surveyed runners (82.1%)—Table 4.

Summarizing, the desire to maintain physical condition and health (82.1%), the desire to develop passion of running (70.9%) and the desire to test yourself (74.6%) were the most often declared motives among runners.

Respondents were also asked which group of motives was the most important for them. Runners could choose only two groups of motives (Table 5).

The most important group of motives was Group B, related to the scope of experience orientation (37.7%) and a group D of motives related to result orientation and competition (36.8%); however, as previous answers showed, it was rather about the desire to test yourself than the desire to win. In third place there was a Group A—motives related to social orientation (26.1%). It turns out that passion for running and care for health and physical condition, although they were reported most frequently by respondents, but they did not matter the most. Through participation in non-elite sport, other functions are more important for the postmodern person, especially social and psychological. The result of this research constitutes a conceptualization of the running event in the dimension of social and psychological interactions which reveal and demonstrate its creative layers and contemporary meaning which has already gone deeply beyond functions of only meeting a need of running or care for health and physical condition.

In order to further analyze the obtained result, it was decided that runners should be asked to define the intensity level of their satisfaction with participation in the 6th Poznań Half Marathon on a 10-point Likert scale (Table 6).

The result of the study turned out to be high (Median = 9). It turned out that as many as 35% of respondents declaring the intensity of satisfaction at the level of 10 points, 9 points were chosen by 23% of respondents, and 8 points by 23.8% of respondents. In addition, 81.8% of those surveyed outlined a range of 7–10 points. The vast majority of the respondents were satisfied with their participation in the event. The event has fulfilled its functions, among others socio-psychological, not just health and passion of running. Kurtzman and Zauhar [45] also pointed out that among the significant motivations of sports consumers there is a “magical atmosphere” full of impressions, sensations, and emotions accompanying, for example, sports events, and interpersonal factors—the need for socialization with other people. Williams adds that the decision to participate in organized sport is always made under the influence of a combination of different factors, not because of one main motive. He claims that the motives of a social, cultural, and psychological nature are very important [46]. It turns out that in addition to performing sports events such basic functions as the possibility of taking care of health and physical condition or the possibility of developing a passion for sports, modern sportsmen also count on issues such as the ability to meet the need to feel strong emotions, and the possibility of a sense of unity and integration with other people. The modern sports event, which is the 6th Poznań Half Marathon, is an important element of the “society of experiences” (see G. Schulze theory). Athletes look for activity “from recreation to excitement” and self-examination (according to the theory of “sensation seeking”—see M. Zuckerman theory (According to the theory of Zuckerman, we observe the importance of experiencing strong emotions associated with participation in a sports event, describing them as sensation-seeking. Researcher, experienced strong emotions related to the mood of a given event, considers one of the most important factors influencing the choice and effectiveness of various forms of sports and recreation activities (Zuckerman, 1994)). This event also becomes a postmodern form of participation in social life—it gives a chance to feel part of a larger human community. As we live now in the culture of individualism, mass sports events become a postmodern form of participation in social life—they allow people to feel part of the community of runners. The need for affiliation is now extremely important in Western societies, which currently do not create many possibilities for collectivist behavior, as in Eastern societies.

The next stage of the research was to examine the differences in motivations for running between the surveyed men and women. The results of the research demonstrate the relevance of specific motives. In the group of motives in the scope of social orientation (Table 7), the most important proved to be the desire to feel unity and integration with other people. This was reported by 49.4% of the surveyed men (171) and 51.4% of women (110).

Belonging to the subculture of runners was the important motive for almost 28.3% of surveyed men (97), while women (63) defined the significance of belonging to the running subculture at 29.4%. Desire to gain recognition in the eyes of others was important for 70 surveyed men (20.2%) and for 52 surveyed women (24.3%). The desire to sense equality during the race was mentioned by almost 19.4% (67) for running men and 24.3% (52) for running women. The statistically significant difference (*p* < 0.05) between these two groups of respondents was found only in one motive: prevailing fashion—that participation in sports events is currently fashionable—selected by 9.2% (32) of men and 17.8% (38) of women (*p =* 0.003). This means that women are more sensitive to current trends in the world of sport. “Cramer’s Phi φ” (Effect Size for Chi-square Test) = 0.12 (small effect).

Among motives in the scope of experience orientation (Table 8) the most important motivation for both groups of respondents was the desire to experience strong emotions that are associated with participation, which was selected by 64.4% men (223) and of 66.3% women (142). The next one, according to the importance, is the desire to have fun, which obtained 52% (180) of surveyed men’s indications and 56.1% (120) of surveyed women’s indicators. In the third place for men was the mood and atmosphere throughout the whole event—47.4% (164). The atmosphere and spirit of the event take the third place with 107 women (50.0%).

Participation in the Half Marathon was important for both groups of respondents (men and women) because they are looking for strong emotions related with it. The statistically significant difference (*p* < 0.05) between these two groups of respondents was found only in the desire to get away from everyday life which indicated more women (27.1%) than men (17.6%), *p =* 0.007. “Cramer’s Phi φ” (Effect Size for Chi-square Test) = 0.11 (small effect).

The research showed that in motives in Group C—within the scope of factual orientation, the most important is the desire to develop a passion connected with running (Table 9) reported by 71.4% (247) of surveyed men and by 70.1% (150) of surveyed women. The next important motive: “I am drawn by the attractiveness of the sports part of the half-marathon” was indicated by 101 men (29.2%) and 50 women (23.4%).

Among the motives within the scope of the result orientation (Table 10), the most often indicated was the desire to test themselves, at the level of 73.4% (254) of men and at the level of 76.6% (164) of women. The motive connected with the desire to achieve the avowed goal was also highly rated—it was indicated by 61.3% (212) of the surveyed runners—men and 57% (122) of women. Participation in sports competition was more important for male runners (142) 41% than for women (75) 35%. The desire to win and high (international) ranking in this sports event was indicated by a small number of the respondents. It turns out that these factors are not important either for men or women.

Both men and women running in the surveyed event were asked about other motives for participation in the half marathon. Most of them marked the desire to maintain good physical condition and health (Table 11). This motive was important for 82.9% (287) of the surveyed men and by 80.8% (173) of the surveyed women.

The respondents were also asked to indicate which group of motives is the most important for them (Table 12).

The major number of men respondents pointed at Group D (36.7%)—the one specifying result orientation, caused by the need to identify with success, and in the case of failure, showing sympathy and solidarity. Motivations in this group are the most serious for the surveyed men. A similar percentage (36.9%) of surveyed women indicated Group D as important. The first place motivation for surveyed women (42.5%) was occupied by Group B motives—including, inter alia, the desire to experience strong emotions associated with participation. Group B was important for surveyed men too, which was indicated by 34.7% of respondents. In the third place in terms of importance for the both groups of respondents was Group A—the one determining the scope of social orientation. Motivations belonging to this group are the desire to feel unity and integration with other people, belonging to the subculture of runners, the desire to sense equality during the race, desire to gain recognition in the eyes of others, and prevailing fashion—participation in sports events because it is currently fashionable. It turns out that for both men (24.3%) and women (28.9%), social factors are important too.

The results of the research showed that women more often than men look for sport in detachment from everyday life, and the most important group of motives turned out to be for them the desire to experience strong, positive emotions. On the other hand, men primarily want to test themselves and their physical capabilities.

## 5. Discussion and Final Conclusions

Motives focused on emotions and social ties confirm that contemporary sport is full of important social and psychological functions, and health and fitness do not count so much for marathoners despite the ideology of healthism (Crawford theory). The popularity of marathons and half marathons fulfills a number of important socio-psychological factors, include enabling people to build a sense of connection and integration with other people, sporting community, becoming a postmodern form of participation in social life. The need to feel strong emotions (in the case of running events often leading to exhaustion of the body) is part of the concept of modern sports activity, which is more often referred to as the transition “from recreation to excitement”. We live in a society of emotions (see theory G. Schulze), and in sport people look for strong feelings (see the sensation seeking theory of M. Zuckerman). Sporting events satisfy the desire to experience strong emotions and psychological needs, which are at the top of hierarchy of post-modern human needs. For active participants of sports events, the desire to win is not the most important. More important is to be able to participate in a sports competition—the willingness to test oneself or the desire to achieve the avowed goal (see serious leisure theory of Stebbins).

According to D. Ross’s research [47], young men with higher education, who are professionally active, are the most active participants of running events in the USA. Data collected during the 6th Poznan Half Marathon, from a sample of 560 runners: 346 men respondents and 214 women who participated in the event voluntarily shows the same tendency in Poland. We checked the socio-demographical profile of runners in Poland: the vast majority of participants were young, with higher a education, The employment status defined by the respondents was very good, because the majority were professionally active or were students. Unfortunately, fewer women than men have been studied. It turns out that still fewer women participate in mass running events. In 2003, Masters & Ogles [48] reported that in their study of motivation among the Midwestern Marathon runners included just over 250 women at more than 1500 participants. The observed trend raises the question of the motives behind marathon runners, particularly due to the fact that in the context of everyday life, both training and running marathons itself are kind of a luxury, because they require a lot of free time on the one side and good health on the other. We may highlight the search for strong emotions and experiences, among others (as we live in an experience-centered society, see G. Schulze theory), which are brought to us through sporting rivalry and the possibility of making social relationships (the need to feel unity during such events) in a period of time where we live in a society of individuals.

The most often indicated motives for both surveyed men and women turned out to be the desire to maintain good physical condition and health (men 82.9%, women 80.8%), the desire to test themselves (men 73.4%, women 76.6%), the desire to develop passion for running (men 71.4%, women 70.1%), and the desire to achieve an avowed goal (men 61.3%, women 57%). Moreover, it turns out that for women, the desire to feel strong emotions associated with participation in sporting event (66.3%), and the desire to have good fun (56.1%) were more often important than for men (66.4% and 52%). Results were similar in the desire to sense the unusual mood during the whole event (women 50%, men 47.4%). All motives in Group B—motives within the scope of experience orientation was more important for women (42.5%) than for men (34.7%). Group D—motives within the scope of result orientation was important for both groups of respondents, especially the desire to participate in sports competition (men 41%, women 35%). What is interesting was that the desire to win was not important for the respondents (men 4%, women 2.3%). For both men and women the most important motivation is the desire to test themselves (result orientation). For men, a bit more important is the desire to maintain physical condition and health. For women a bit more important are social relations (social orientation) and emotions related to participation in running event. These results from the Polish running event correlate with Yates’ research [36], who also concludes that men are judged of their physical effectiveness or strength, and with Summers and co-authors research [38], who claims that women report greater benefits from running than men in terms of opportunities to meet people, relief from depression, and feeling less shy.

In general, all respondents reported that the need to experience strong emotions related to participation was very important for them. Both groups of surveyed respondents received adrenaline, pleasure, relaxation, and the escape from the duties and hardships of everyday life. The last issue is especially important for women. Results shows that people participate in running events not only for physical activity, but also for mental health.

The significant difference between surveyed men and women appeared in Group B, the desire to get away from everyday life turned out to be more important for women (27.1%) than for men (17.6%) and in Group A, for women (17.8%) was more important prevailing fashion (participation in sports events is currently fashionable) than for men (9.2%). It confirms the fact that women usually have different lifestyles, and find everyday life less interesting (home and family duties). That is why sports competition is important to them. The main results of the study of Ogles and co-authors [32] from 1995 shows that women endorsed weight concern, affiliation, self-esteem, psychological coping, and life meaning as more important motives for running than men. 

Marathon running is a leisure behavior that has seen tremendous growth during the past decade [49], also in Poland. The research has shown that running events in this country have significant psychological and social impact on the participants. Moreover, people practicing running as progressive recreation and taking part in half marathons demonstrate a high demand for stimulation (sensation seeking). In general, results show that people participate in running events not only for physical activity, but also for mental health. They receive strong emotions, adrenaline, and pleasure. These results might be useful for sport managers and city government to analyze consumer behavior and utilize the results in the strategic, sustainable planning, marketing, and implementation of physical culture and leisure in connection with mass sport events’ organization. Because of these known benefits associated with participation in running events, it is important to continue promoting participation in such events. In order to optimize promotional strategies and tailor them towards population subgroups that are currently underrepresented in running events, it is necessary to gain more detailed insight into the profile of participants. Previous studies in the domain of running events focused almost exclusively on characteristics of participants [20,21,22,23] while motivations for running in sporting events are not recognized in more detail.

## Figures and Tables

**Table 1:** Socio-demographic characteristics of respondents.

Socio-Demographic Characteristics	Men (*n* = 346)		Women (*n* = 214)		All (*n* = 560)	
	*n*	%	*n*	%	*n*	%
Age						
<18	7	2.0	6	2.8	13	2.3
19–25	136	39.3	89	41.6	225	40.2
26–35	141	40.8	87	40.7	228	40.8
36–50	46	13.3	23	10.7	69	12.3
51–70	16	4.6	9	4.2	25	4.5
Education level						
Primary education	6	1.7	2	0.9	8	1.4
Vocational education	8	2.3	5	2.3	13	2.3
Secondary education	103	29.8	56	26.2	159	28.4
Incomplete higher education	67	19.4	45	21.0	112	20.0
Completed higher education	162	46.8	106	49.5	268	47.9
Employment status						
School pupil (<18 years)	12	3.5	12	5.6	24	4.3
Student	105	30.3	73	34.1	178	31.8
Professionally active	202	58.4	116	54.2	318	56.8
Unemployed	13	3.8	7	3.3	20	3.6
Pensioner	14	4.0	6	2.8	20	3.6

**Table 2:** Motives within the scope of social and experience orientation (*n* = 560).

Groups of Motives	All (*n* = 560)	%
*Group A* *Motives within the scope of social orientation*		
Desire to feel unity and integration with other people	281	50.2
Belonging to the subculture of runners	160	28.6
Desire to sense equality during the race	119	21.2
Desire to gain recognition in the eyes of others	122	21.8
Prevailing fashion—participation in sports events is currently fashionable	70	12.5
*Group B* *Motives within the scope of experience orientation*		
Desire to have good fun	300	53.6
Desire to experience strong emotions associated with participation	365	65.2
Desire to sense the unusual mood during the whole event	271	48.4
Desire to have enjoyable leisure time/entertainment	149	26.6
Desire to express happiness, e.g., resulting from winning/success	97	17.3
Desire to get away from everyday life	119	21.2
I am allured by the attractiveness of the city in which the event takes place	31	5.5

**Table 3:** Motives within the scope of factual and result orientation (*n* = 560).

Groups of Motives	All (*n* = 560)	%
*Group C* *Motives within the scope of factual orientation* (*connected with running*)		
Desire to develop passion (running)	397	70.9
I am drawn by the attractiveness of the sports part of the half-marathon	151	27.0
I am drawn by the attractiveness of the extensive program of events accompanying	63	11.3
*Group D* *Motives within the scope of result orientation*		
Desire to test yourself	418	74.6
Desire to achieve the avowed goal	336	59.6
Desire to participate in sports competition	217	38.8
Desire to win	12	2.1
High (international) rank of this sporting event	19	3.4

**Table 4:** Other motives (*n* = 560).

Groups of Motives	All (*n* = 560)	%
*Group E* *Other motives*		
The desire to maintain physical condition and health	460	82.1

**Table 5:** Significance of particular groups of motives covered by the research (*n* = 560).

Significance of the Motives Group	All	%
Group A *Motives within the scope of social orientation*	146	26.1
Group B *Motives within the scope of experience orientation*	211	37.7
Group C *Motives within the scope of factual orientation*	60	10.7
Group D *Motives within the scope of result orientation*	206	36.8
Group E *Other* (*The desire to maintain good physical condition and health*)	115	20.5

**Table 6:** Study of the level of satisfaction with the participation in the 6th Poznan Half Marathon.

Mean	Median	Minimum	Maximum	Standard Deviation
8.6	9	2	10	1.4

**Table 7:** Motives within the scope of social orientation.

Groups of Motives	Men (*n* = 346)		Women (*n* = 214)		All (*n* = 560)	*p*
	*n*	%	*n*	%		
*Group A* *Motives within the scope of social orientation*						
Desire to feel unity and integration with other people	171	49.4	110	51.4	281	0.649
Belonging to the subculture of runners	97	28.3	63	29.4	160	0.720
Desire to sense equality during the race	67	19.4	52	24.3	119	0.165
Desire to gain recognition in the eyes of others	70	20.2	52	24.3	122	0.257
Prevailing fashion—participation in sports events is currently fashionable	32	9.2	38	17.8	70	0.003

**Table 8:** Motives within the scope of experience orientation.

Groups of Motives	Men (*n* = 346)		Women (*n* = 214)		All (*n* = 560)	*p*
	*n*	%	*n*	%		
*Group B* *Motives within the scope of experience orientation*						
Desire to have good fun	180	52.0	120	56.1	300	0.350
Desire to experience strong emotions associated with participation	223	64.4	142	66.3	365	0.645
Desire to sense the unusual mood during the whole event	164	47.4	107	50.0	271	0.549
Desire to have enjoyable leisure time/entertainment	93	26.9	56	26.2	149	0.853
Desire to express happiness, e.g., resulting from winning/success	62	17.9	35	16.4	97	0.634
Desire to get away from everyday life	61	17.6	58	27.1	119	0.007
I am allured by the attractiveness of the city in which the event takes place	19	5.5	12	5.6	31	0.953

**Table 9:** Motives within the scope of factual orientation.

Groups of Motives	Men (*n* = 346)		Women (*n* = 214)		All (*n* = 560)	*p*
	*n*	%	*n*	%		
*Group C* *Motives within the scope of factual orientation*						
Desire to develop my passion (running)	247	71.4	150	70.1	397	0.743
I am drawn by the attractiveness of the sports part of the half-marathon	101	29.2	50	23.4	151	0.131
I am drawn by the attractiveness of the extensive program of accompanying events accompanying	37	10.7	26	12.1	63	0.596

**Table 10:** Motives within the scope of result orientation.

Groups of Motives	Men (*n* = 346)		Women (*n* = 214)		All (*n* = 560)	*p*
	*n*	%	*n*	%		
*Group D* *Motives within the scope of result orientation*						
Desire to test yourself	254	73.4	164	76.6	418	0.393
Desire to achieve the avowed goal	212	61.3	122	57.0	336	0.317
Desire to participate in sports competition	142	41.0	75	35.0	217	0.157
Desire to win	14	4.0	5	2.3	13	0.277
High (international) rank of this sports event	8	2.3	4	1.9	18	0.725

**Table 11:** Other motives.

Groups of Motives	Men (*n* = 346)		Women (*n* = 214)		All (*n* = 560)	*p*
	*n*	%	*n*	%		
*Group E* *Other motives*						
The desire to maintain physical condition and health	287	82.9	173	80.8	460	0.527

**Table 12:** Significance of particular groups of motives covered by the research.

Significance of the Motives Group	Men (*n* = 346)		Women (*n* = 214)		All	*p*
	*n*	%	*n*	%		
Group A *Motives within the scope of social orientation*	84	24.3	62	28.9	146	0.218
Group B *Motives within the scope of experience orientation*	120	34.7	91	42.5	211	0.062
Group C *Motives within the scope of factual orientation*	37	10.7	23	10.7	60	0.983
Group D *Motives within the scope of result orientation*	127	36.7	79	36.9	206	0.959
Group E *Other* (*The desire to maintain physical condition and health*)	77	22.2	38	17.7	115	0.200
